# Assessing wastewater-based epidemiology for the prediction of SARS-CoV-2 incidence in Catalonia

**DOI:** 10.1038/s41598-022-18518-9

**Published:** 2022-09-05

**Authors:** Bernat Joseph-Duran, Albert Serra-Compte, Miquel Sàrrias, Susana Gonzalez, Daniel López, Clara Prats, Martí Català, Enric Alvarez-Lacalle, Sergio Alonso, Marina Arnaldos

**Affiliations:** 1 CETAQUA Water Technology Center, Cornellà de Llobregat, Catalonia Spain; 2 Department of Physics, Universitat Politècnica de Catalunya (UPC-BarcelonaTech), Barcelona, Catalonia Spain; 3 Nuffield Department of Orthopaedics, Rheumatology, and Musculoskeletal Sciences (NDORMS), University of Oxford, Oxford, UK

**Keywords:** Infectious diseases, Applied mathematics

## Abstract

While wastewater-based epidemiology has proven a useful tool for epidemiological surveillance during the COVID-19 pandemic, few quantitative models comparing virus concentrations in wastewater samples and cumulative incidence have been established. In this work, a simple mathematical model relating virus concentration and cumulative incidence for full contagion waves was developed. The model was then used for short-term forecasting and compared to a local linear model. Both scenarios were tested using a dataset composed of samples from 32 wastewater treatment plants and severe acute respiratory syndrome coronavirus 2 (SARS-CoV-2) incidence data covering the corresponding geographical areas during a 7-month period, including two contagion waves. A population-averaged dataset was also developed to model and predict the incidence over the full geography. Overall, the mathematical model based on wastewater data showed a good correlation with cumulative cases and allowed us to anticipate SARS-CoV-2 incidence in one week, which is of special relevance in situations where the epidemiological monitoring system cannot be fully implemented.

## Introduction

Infectious disease outbreaks can have negative effects on society, especially in dense urban areas^1^. Since 2000, several epidemics caused by viruses have occurred worldwide, such as H1N1, Ebola, Zika, Middle East Respiratory Syndrome (MERS) and Severe Acute Respiratory Syndrome (SARS)^2^. Traditional strategies for surveillance of disease include diagnostic analysis, clinical samples, questionnaire surveys, national census, and previous literature, among others^2,3^. However, these methodologies may require considerable labour and high cost, and they are time-consuming. In addition, the reported results are not available in real time, and they lack the ability to predict critical locations and moments for disease onset^1^. Therefore, there is a need for strategies that allow early warning within epidemiological surveillance.

In this scenario, wastewater-based epidemiology (WBE) has emerged as a potential strategy for epidemiological surveillance, providing objective and comprehensive analysis, as well as real-time monitoring of the health status of a given population^2^. The WBE approach is based on the fact that wastewater contains a wide range of chemical and biological compounds derived from human activity^4^. Furthermore, some viruses can be present in the excreta of infected people and end up in wastewater; hence, wastewater sampling could be representative of community-based urine and faecal sampling^1^. Viruses do not replicate outside of the host cell, and the viral levels present in wastewater would theoretically be related to the concentrations excreted by the corresponding population^5^. Nevertheless, the complexity of the wastewater matrix should be considered as it may affect virus particle concentration (due to dilution effects) or integrity (because of detergents occurrence).

Wastewater monitoring for surveillance of virus outbreaks was initially used to trace poliomyelitis epidemics in the 1950s and 1960s^6,7^. Later, several studies demonstrated the occurrence of different viruses in the excreta of infected people and in wastewater, including adenoviruses, enteroviruses, astroviruses, hepatitis A and E viruses, noroviruses, rotaviruses and coronaviruses, among others^1^. In addition to virus detection, previous studies have traced the circulation of norovirus within populations through molecular analysis in wastewater^8^. In that study, a novel norovirus variant outbreak was identified, and the authors postulated the need for future long-term sewer surveillance of norovirus and potentially other human viruses.

The application of WBE has been suggested for coronavirus disease 2019 (COVID-19), caused by the SARS-CoV-2 virus, which was declared an epidemic by the World Health Organization (WHO) in March 2020. SARS-CoV-2 is mainly transmitted through small respiratory droplets or aerosols of infected people^9^. Accordingly, SARS-CoV-2 has been reported in the respiratory tract and blood of infected people^10^. In addition, SARS-CoV-2 infection has also been detected in gastric and rectal epithelial cells; hence, the release of virus particles from patients’ faeces, including both symptomatic and asymptomatic patients, has been shown^11^.

The occurrence of virus particles in faeces from infected people led to the detection of SARS-CoV-2 RNA in wastewater^12–17^. This was found to be a useful source of information to track pandemic dynamics and characteristics. For instance, sequencing of the SARS-CoV-2 genome in wastewater can provide information concerning the circulation of variants in the target city or community^18,19^. In addition, several studies determined a correlation between SARS-CoV-2 RNA in wastewater and reported COVID-19 cases, which highlighted the potential application of sewage surveillance to study spatial and temporal variations in virus circulation in the population^20,21^. The number of individuals infected by SARS-CoV-2 has also been estimated using the quantification of viral gene copies per litre and considering the viral particle shedding per individual^22,23^.

One particularly intriguing aspect of SARS-CoV-2 RNA detection in wastewater is its potential capacity to anticipate virus outbreaks within the population^24,25^, as well as to estimate the SARS-CoV-2 prevalence^26,27^. Some studies detected SARS-CoV-2 RNA in wastewater from areas with a low prevalence of COVID-19, which highlighted the potential application of WBE as an early warning system^12,17^. The predictive capacity of WBE has been already explored through different modelling approaches. Compartmental SEIR-type models (S standing for Susceptible, E for Exposed, I for Infectious and R for Recovered) have been adapted to include wastewater RNA determinations^28,29^. These models allow for an extremely informative mechanistic approach; nevertheless, they entail the determination of several parameters that make their generalisation difficult to other sites. Different empirico-statistical models have also been explored to estimate the COVID-19 prevalence in two Greek municipalities^30^ as well as to explore the prediction capacity of the incidence dynamics in specific communities like Sendai city in Japan^31^, Oklahoma city in USA^32^, and four Austrian communities^33^. These approaches are focused on particular contexts where the relationship between the area of influence of the wastewater plants and the epidemiological dynamics in this area can be straightforward and homogeneous. Some of these models explore the lag between wastewater determinations and incidence variables, giving rise to values between 2 and 7 days^32,33^. Melvin et al.^27^ propose the use of the pepper mild mottle virus (PMMoV) concentration in human faeces to standardise the concentration of SARS-CoV-2 in wastewater samples in the state of Minnesota (USA) and estimate the lag between these determinations and the population level incidence, which is found to be in a window of 15–17 days.

This paper explores the predictive potential of wastewater analyses as a sentinel for changes in epidemiological trends and anticipation of their changes in magnitude in a heterogeneous region of 7.7 million inhabitants, as well as its viability as a method. A methodology was developed to use SARS-CoV-2 RNA detection in wastewater to anticipate COVID-19 dynamics in populations to be applied as an early warning system. For the mathematical model development, data from the influent of 32 wastewater treatment plants (WWTPs) in Catalonia (Spain) were considered. The model is built from an empirico-statistical approach that guarantees a feasible parameterisation process, as well as its usability in different contexts. After model implementation, it was evaluated for its ability to predict COVID-19 incidence in the corresponding areas within one week.

## Materials and methods

### Epidemiological context and data

Catalonia is a region with more than 7.7 million inhabitants in northeastern Spain. It is divided into 9 health regions, which are split into several basic health areas (BHAs), each of which is the area of influence of a primary care centre. From September 2020 to March 2021, Catalonia suffered two COVID-19 waves, with different intensities depending on the region. Overall, there was a wave that started at the end of September and peaked in the last week of October 2020 (autumn wave), which was followed by a new wave that started the second week of December and peaked the second week of January (winter wave). The maximum 14-day cumulative incidences were approximately 800 and 700 cases per 100,000 inhabitants, respectively. Both were mainly controlled by means of nonpharmacological interventions (NPIs) of different intensities, focused on the closure of and restrictions on specific economic activities (i.e., restaurants and shopping centres, among others) and restrictions on mobility (municipal or county level).

During this period, the monitoring of the epidemiological situation was based on health data, such as COVID-19 positive diagnostics, admissions to hospitals and intensive care units, COVID-19-caused fatalities, among others. These data are updated daily in open portals^34^ and given with different granularities with respect to health regions, age and sex. In general, there is a delay of approximately 3–4 days until their consolidation, meaning that the data of a certain day are consolidated after this period. Usually, the date assigned to a case corresponds to the day when the sample was obtained, although the infection certainly occurred a few days before^35^. Therefore, when a change in the trend is detected in the reported data, this is the consequence of a change in the infection trend that occurred a few days before.

For this study, freely available^36^ daily new cases per basic health area during the study period (September 2020–March 2021) were used. These data were used to assess the cumulative incidence as explained below.

### WWTP data

SARS-CoV-2 RNA concentrations in wastewater were obtained from a previous study^37^. The data of the study are publicly available at the online visualization platform^38^. This platform was boosted by the Catalan Public Health Agency and the Catalan Water Agency, providing information on SARS-CoV-2 RNA occurrence at the influent of different WWTPs located in Catalonia (N. E. Spain).

To automate the data update process, the repository published by the same authors was accessed through GitHub^39^. While performing this study, a series of Python^40^ codes were programmed to always have the latest data of the presence of viruses and the status of the WWTPs.

Next, the characteristics of the samples used for the determination of SARS-CoV-2 RNA are briefly described based on the information from a previous study^37^.

The data gathered considered SARS-CoV-2 RNA concentrations from water samples taken at the influent of 32 different WWTPs. Despite the study of Corominas et al.^37^, which reported data for 56 plants, only WWTPs where a weekly measure of SARS-CoV-2 was reported were considered in the present work. The plant characteristics are summarized in Table 1 of the Supplementary Material. The considered WWTPs covered different plant sizes, from a design capacity of 18,450 to 3,000,000 equivalent inhabitants. In combination, the plants treat wastewater generated from more than 5 million equivalent inhabitants. In addition, the plants covered zones with different degrees of industrialization as well as with a different touristic pressure.

Wastewater samples were obtained at the influent of each of the 32 WWTPs from September 2020 until March 2021. Overall, 952 untreated wastewater samples were considered in the present work. Sampling was performed by applying automatic samplers to obtain 24-h-composed-flow proportional water samples in most cases. However, due to the instrumentation availability, samples were obtained as 8 h or 12 h integrated samples from some of the plants (Table 1 of Supplementary Material). After sampling, raw sewage samples were transported under cooling conditions to one of the three different laboratories involved in the study described in Corominas et al.^37^ The samples were analysed within 24 h after sampling through the analytical process for SARS-CoV-2 quantification in wastewater described in Corominas et al.^37^. The analysis included sample concentration, RNA extraction and SARS-CoV-2 RNA quantification through reverse transcription quantitative polymerase chain reaction (RT–qPCR). A minimum of two specific genetic targets for SARS-CoV-2 were used in all applied RT–qPCR protocols, such as N1/N2 or N1/IP4. The highest concentration detected from the different targeted genes was used for each positive sample for further data processing.

### Data analysis

The objective of the data analysis was to establish a functional relationship between the measured SARS-CoV-2 RNA concentrations in WWTP samples and the corresponding cumulative incidence of notified cases. Variations in the concentrations were expected to anticipate variations in the cumulative incidence because infected individuals would start to contribute SARS-CoV-2 RNA to wastewater days before they were notified to the public health system. A functional relationship taking into account this delayed behaviour should allow for short-term forecasts of the variations in cumulative incidence, thus providing an early warning tool for infection outbreaks. The relationship between concentration and cumulative incidence was studied at WWTPs and associated BHAs levels and globally using a population-weighted average of the two variables.

The first step of the data treatment was to homogenize the reporting frequencies of concentration from the different WWTP laboratories and the notified cases from the different BHAs. Concentration data may have come as weekly or biweekly measurements, while cumulative notified cases were reported daily. In both cases, however, there may have been occasional missing records that had to be inferred by interpolation.

Since cumulative cases were reported daily, missing data in these time series usually consisted of only a few days and therefore were interpolated by linear interpolation.

Daily cumulative cases were used to compute the cumulative incidence over $n$ days ${I}_{n}$ [cases per 10^5^ inhabitants] as follows:

1 $$\begin{array}{*{20}c} {I_{n} \left( t \right) = C\left( t \right) - C\left( {t - n + 1} \right)} \\ \end{array}$$

where $C(t)$ was the daily notified cumulative cases per 10^5^ inhabitants for day $t$. Although the $n=14$ days cumulative incidence is a common variable in epidemiology, a value of $n=21$ days was used in this work since it results in larger delays with respect to virus concentrations, allowing for longer-term forecasts.

**Figure 1:**
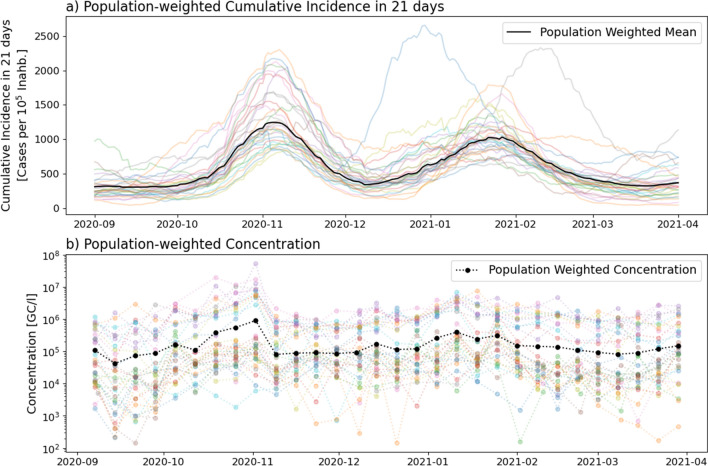
Population-averaged 21-day cumulative incidence [cases per 10^5^ inhabitants] and concentration [GC/l]. Transparent coloured lines show the data corresponding to each BHA and WWTP, and the black lines show the population-weighted averages.

As mentioned above, concentration data have a weekly frequency. To operate these concentration data together with the daily cumulative case data, it was necessary to up-sample it to daily frequency by means of interpolation techniques. Daily frequency also allowed the use of delays in the order of days when adjusting the concentration-cumulative incidence relationship. In addition to up-sampling, smoothing of the concentration signal also appeared to be necessary. It was observed that consecutive weekly measurements of virus concentration may show very big variations, sometimes of orders of magnitude (Fig. 1b). These oscillations do not have a correspondence in the cumulative incidence data and therefore must be attenuated by some smoothing technique before trying to establish any relationship between the two variables.

The locally weighted scatterplot smoothing (LOWESS)^41^ technique was selected to pre-process the virus concentration data since it could be used to both interpolate and smooth it. A simple Python implementation of the LOWESS method for a Gaussian weight function allowing for interpolation of time-series data was developed for this study. In the following, the LOWESS-interpolated concentration will be denoted by $\rho$ [gene copies per litre, GC/l].

In addition to individual WWTPs and corresponding BHA information, a model of the global evolution of the pandemic over the whole region was developed. To this end, global population-averaged aggregates of both cumulative incidence and virus concentration were computed as follows:

2 $$\begin{array}{*{20}c} {I_{n}^{g} \left( t \right) = \frac{1}{{P_{{{\text{tot}}}} }}\mathop \sum \limits_{i = 1}^{{n_{{{\text{WWTP}}}} }} P_{i} \cdot I_{n}^{i} \left( t \right)} \\ \end{array}$$

3 $$\begin{array}{*{20}c} {\rho_{{}}^{g} \left( t \right) = \frac{1}{{P_{{{\text{tot}}}} }}\mathop \sum \limits_{i = 1}^{{n_{{{\text{WWTP}}}} }} P_{i} \cdot \rho_{{}}^{i} \left( t \right)} \\ \end{array}$$

where $I_{n}^{g}$ [cases per 10^5^ inhabitants] and $\rho_{{}}^{g}$[GC/l] are the global population-averaged cumulative incidence and virus concentration, $I_{n}^{i}$[cases per 10^5^ inhabitants] and $\rho_{{}}^{i}$[GC/l] the cumulative incidence and virus concentration associated with WWTP $i$, $P_{{{\text{tot}}}}$[inhabitants] the total population, and $P_{i}$[inhabitants] the population associated with WWTP $i$.

Figure 1 shows the resulting global variables over the individual WWTP variables.

### Mathematical models

#### Wave model

The following fitting function was used to determine the functional relationship between concentration $\rho$ and cumulative incidence $I_{n}$ along a whole pandemic wave, including an increase and decrease in cases over several weeks:

4 $$\begin{array}{*{20}c} {I_{n} \left( {t + \tau } \right) = a \cdot \left( {\rho \left( t \right) - \rho_{0} } \right)^{b} + I_{n,0} } \\ \end{array}$$

Here, $a, b, {I}_{n,0} ,{\rho }_{0}$ and $\tau$ are fitting parameters to be determined by minimizing the coefficient of determination ${R}^{2}$ between the cumulative incidence data and model-generated samples.

**Figure 2:**
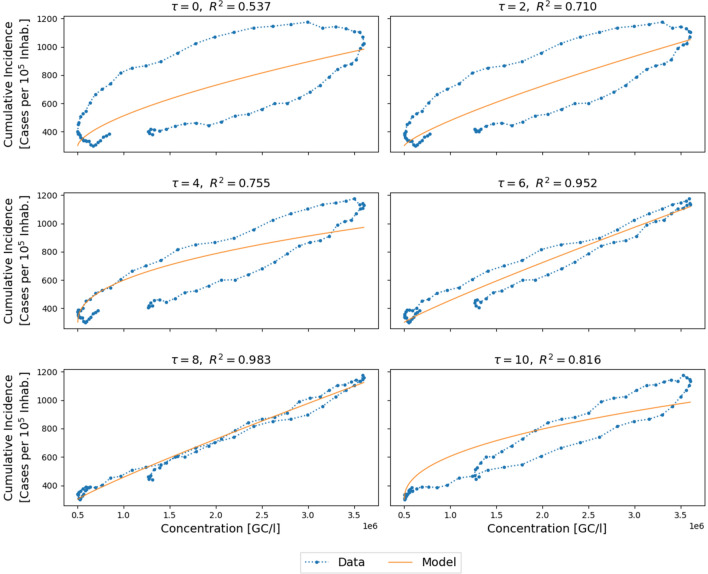
Cumulative incidence as a function of concentration of genetic copies for different values of the delay parameter $\tau$ [days] together with the corresponding fitted functions and ${R}^{2}$ scores. The blue dotted line shows the interpolated cumulative incidence and the smoothed concentration data, and the orange solid line shows the adjusted model. Data correspond to WWTP22 (Rubí) during the autumn wave period.

As mentioned above, infected individuals are known to contribute SARS-CoV-2 genetic material to wastewater days before any illness symptoms appear, and the case is reported to health authorities. Parameter $\tau$ [days] represents an approximation of this phenomenon in terms of cumulative incidence. Since $\tau$ can take only integer values, to estimate it, the previous function fitting was executed several times by applying increasing values of $\tau$ in the measurement data, and the best fitting combination of delay and corresponding parameters $a, b, I_{n,0} , \rho_{0}$ was chosen, as shown in Fig. 2.

Parameter $\rho_{0}$ [GC/l] can be interpreted as a minimum concentration value below which measurements are not reliable and parameter $I_{n,0}$ [cases per 10^5^ inhabitants] as the cumulative incidence corresponding to $\rho_{0}$. Finally, $a$ and $b$ are model parameters that account for the increase in cumulative incidence corresponding to a given increase in concentration.

Different options for the wave model were tested in addition to the function in Eq. 4. The basic requirement after visual inspection of several concentration-cumulative incidence scatter plots was that they should be concave functions with interpretable parameters that could provide some insight about the process and show potential to be generalized to new waves. A more general function could lead to better fitting in some sites at the expense of losing this interpretability. Results for sites with poor fitting are interpreted as sites that behave according to complex dynamics beyond the scope of the study such as geographical mobility of the population among different BHAs.

The optimal parameters for the fitting function according to the ${R}^{2}$ score objective have been obtained as the result of an optimization problem implemented in Python by means of the *minimize* function of the Scikit-learn module^42^.

As discussed in the Results section, this function has been shown to provide a good relationship for a period of time covering a whole contagion wave but not necessarily for a short-term forecast.

#### Short-term forecast models

To assess the potential of the proposed model for short-term forecasting and early detection of disease outbreaks, a successive weekly fit-and-predict approach was simulated using only the data that would be available at prediction time. This procedure mimics the real situation in which every time a new concentration measurement is available, the model is fitted and run to provide an estimation of the near-future behaviour of the cumulative incidence. This fit-and-predict methodology was run for all the studied WWTPs, individually and as an ensemble, and each week in the period from the 1st of October 2020 to the 1st of April 2021.

Two different approaches were evaluated for short-term forecasts. In the first one, the wave model (Eq. 4) was fitted to the concentration and cumulative incidence data since the start of the wave and used to predict the cumulative incidence in the following days based on the model delay. Note that with this approach, the fitting data window increases every time a new prediction is computed. The idea behind this approach is that the power function described in the previous section may provide a good fit for the complete wave, including the rising and falling limbs of the curves, but not necessarily for local short-term behaviour. As a result of this observation, a second forecast approach based on a short fitting window was also used. In this case, a simple linear regression (with delay) was fitted over a sliding backwards window of 2 weeks from the prediction date.

5 $$\begin{array}{*{20}c} {I_{n} \left( {t + \tau } \right) = a\cdot\rho \left( t \right) + b} \\ \end{array}$$

Polynomials of higher degree were also tested but were discarded since they tend to increase rapidly outside the fitting interval resulting into big estimation errors of cumulative incidence. Again, the optimal fitting delay was used to predict the cumulative incidence evolution for the next few days.

#### Model evaluation

Two different indicators were used to evaluate the goodness of fit of each of the models described above. The coefficient of determination ${R}^{2}$ was used to evaluate the model fitting, including the whole wave model and the two approaches for short-term forecasting. The mean absolute percentage error (MAPE) was used to compute the prediction error in the multiple short-term forecasts.

## Results

### Wave model

#### Individual WWTP data results

**Figure 3:**
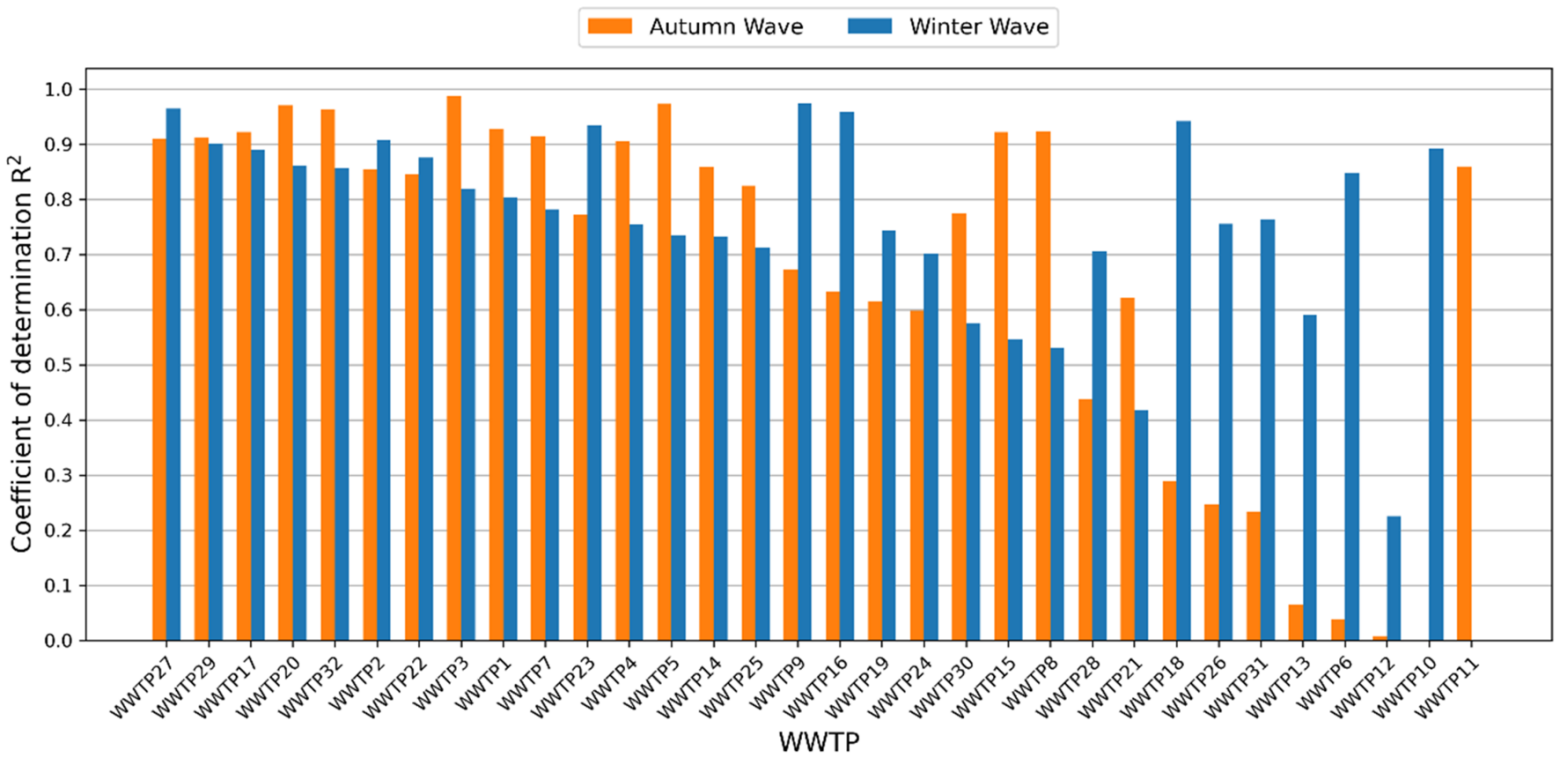
${R}^{2}$ score of the wave model for the different studied WWTPs and the two contagion waves (sorted by the minimum value of the two waves).

Wave model fitting results show different behaviours depending on the particular WWTP and associated BHAs. While 15 out of the 32 WWTPs had an ${R}^{2}$ fitting score greater than 0.7 for both the autumn and winter waves, other WWTPs showed very poor fitting for one of the two waves or even for both (Fig. 3).

**Figure 4:**
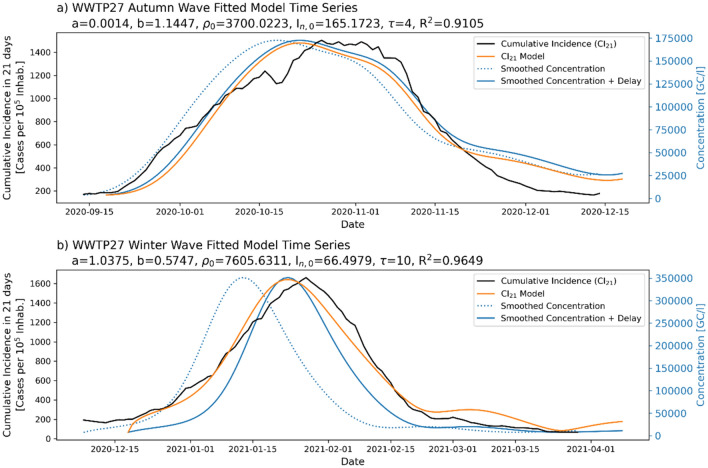
Fitted model for WWTP27 (Tortosa-Roquetes) for the two studied contagion waves. The blue dotted line shows the smoothed concentration, the solid blue line shows the concentration after applying the optimal delay, the black solid line shows the interpolated incidence, and the orange line shows the optimal fitted incidence model.

The highest agreement was found in WWTP27 (Tortosa-Roquetes), with a very good fitting for both waves with ${R}^{2}$ scores over 0.9 (Fig. 4).

#### Global population-averaged data results

**Figure 5:**
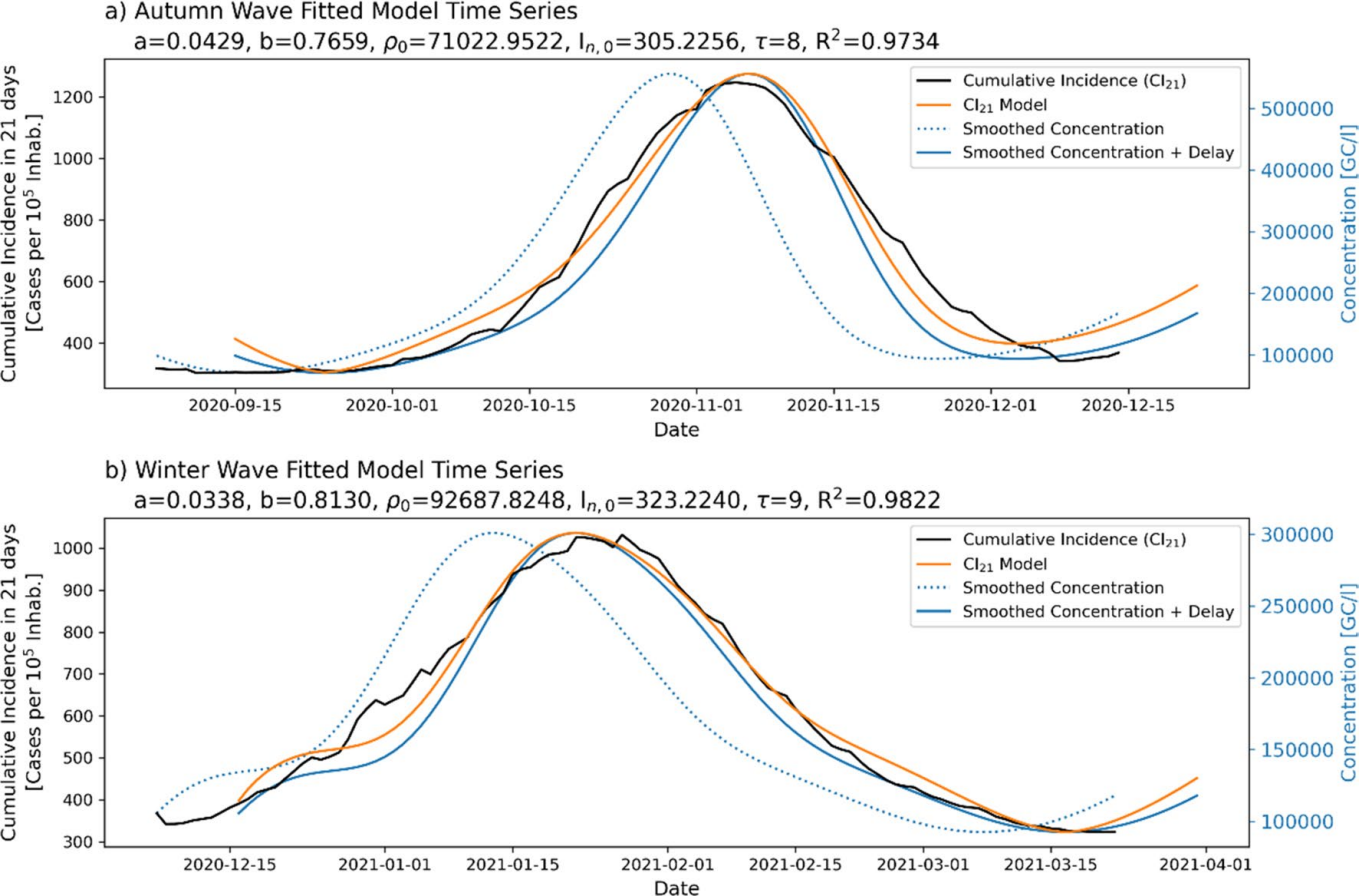
Fitted model for the global population-averaged dataset for the two studied contagion waves. The blue dotted line shows the smoothed concentration, the solid blue line shows the concentration after applying the optimal delay, the black solid line shows the interpolated incidence, and the orange line shows the optimal fitted incidence model.

**Figure 6:**
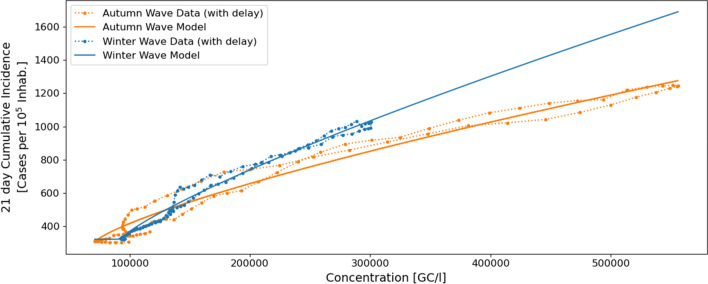
Fitting functions of the wave model for the global population-averaged dataset corresponding to the autumn (orange) winter (blue) waves. The dotted lines show the smoothed concentration-incidence relationship after applying the optimal delay, and the solid lines show the adjusted model.

The wave model had very good fitting results for the population-averaged cumulative incidence and virus concentration in WWTP water, as shown in Fig. 5. The autumn wave showed a ${R}^{2}$ fitting score of 0.97, and the winter wave showed a score of 0.98. An important feature of the fitted models is that they were very similar (Fig. 6), pointing to the idea that they could be used for the prediction of new contagion waves.

These results suggest that the population-averaged dataset is able to compensate for the population mobility fluctuations among the different geographic areas and corresponding WWTPs and BHAs. The resulting global-scale model is therefore more robust than those obtained for individual sites.

### Short-term forecast

As described in the previous sections, two different approaches were tested for the short-term forecast of cumulative incidence: the whole-wave model and a 2-week linear model. Note that for short-term forecasts, data of the complete contagion wave were not available. Therefore, the forecast models were fitted and run with weekly frequency every time a new concentration measurement was available.

For the wave model, the time series corresponding to each WWTP and BHAs were split according to the contagion waves based on the minimum cumulative incidence values of September 2020, December 2020 and March 2021. The model was then readjusted weekly using an increasing dataset including data from the wave start until the last concentration measurement. For the linear model, a fixed 2-week backwards window since the last concentration measurement was used.

**Figure 7:**
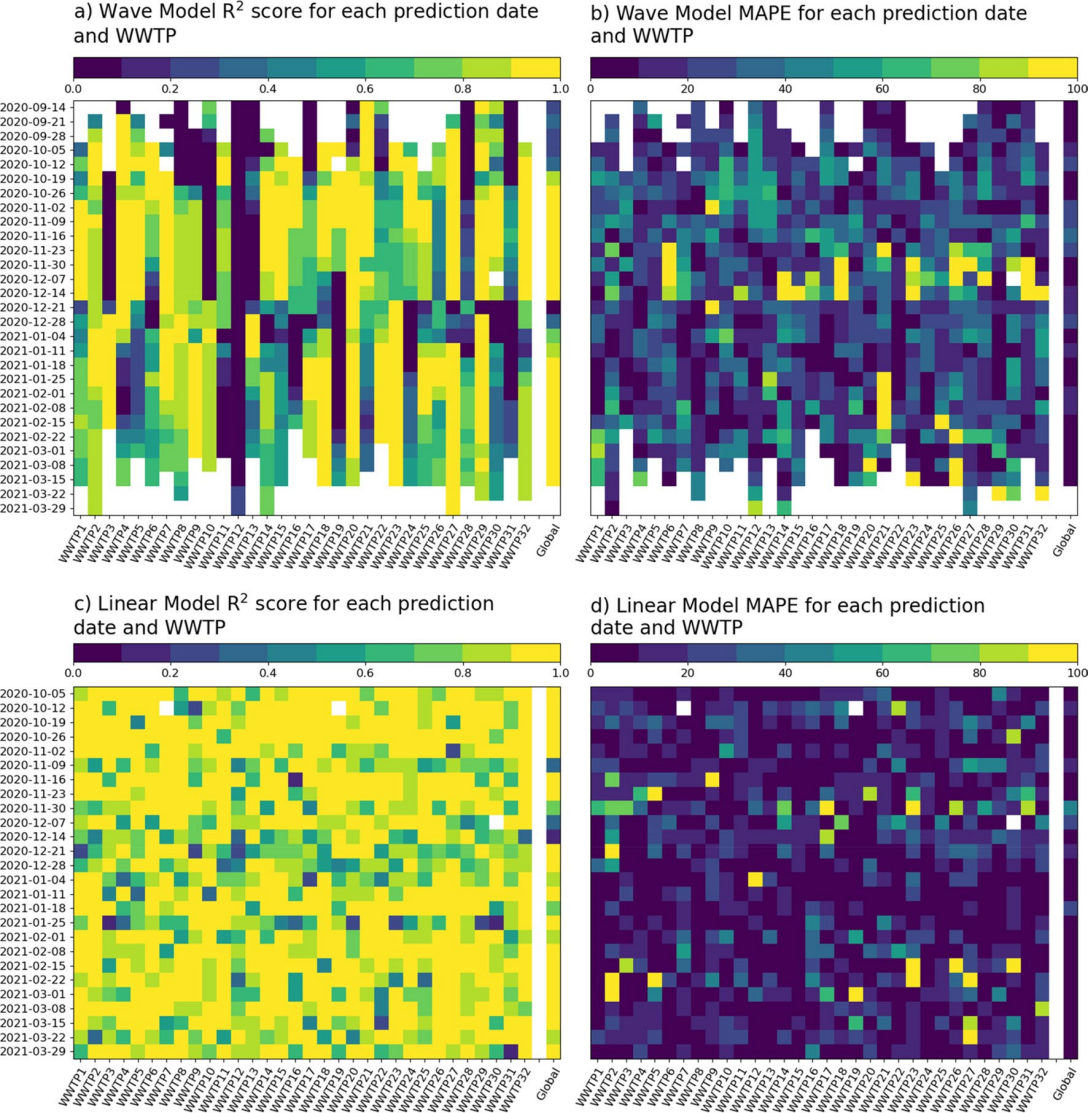
${R}^{2}$scores (**a** and **c**) and MAPE (**b** and **d**) of the Wave Model with increasing fitting window forecasts (**a** and **b**) and the Linear Regression with sliding 2-week fitting window (**c** and **d**) for each WWTP and prediction date. Note that good performance is related to a yellow colour in the ${R}^{2}$ heatmaps (high correlation) and a blue colour in the MAPE heatmap (low error).

**Figure 8:**
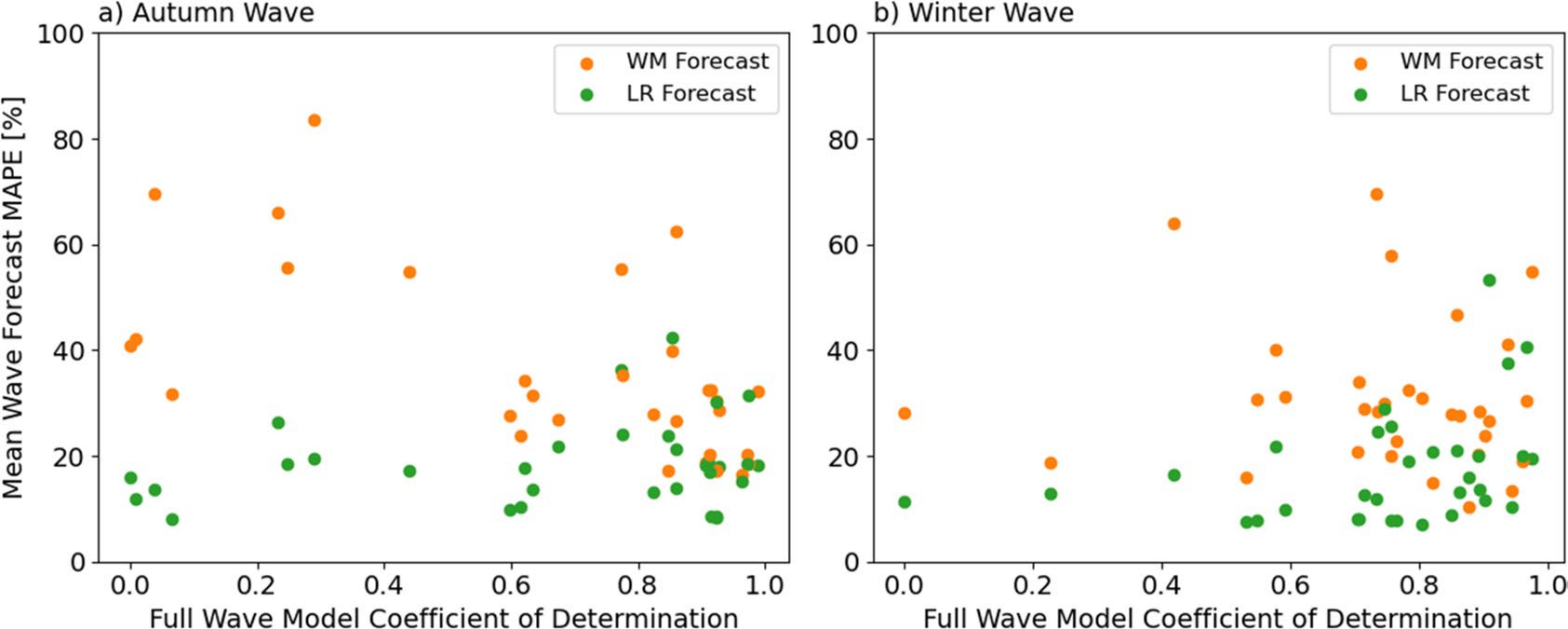
Relationship between the Wave Model coefficient of determination for each WWTP and full contagion wave and the respective mean MAPE for each short-term forecast method (Wave Model with increasing fitting window and linear model with 2-week fitting window). The short-term forecast linear model performed better for those sites where the Wave Model of the full contagion wave showed a low coefficient of determination.

Figure 7 summarizes the results of the fit-and-predict approach for the two models for the different prediction dates and WWTPs. The linear model with a fixed fitting window performed better for short-time forecasts than the wave model. This is explained by the fact that the linear model captured the local dynamics better than the wave model, which needed to adjust to a larger time window. Moreover, the linear model produced better forecasts in WWTPs and contagion waves where the full wave model performed poorly, as shown in Fig. 8.

## Discussion and conclusions

The analysis of wastewater has been found to allow the detection of viruses and variants^18,19^ and can therefore play a role in epidemiological surveillance. In the present work, the viability of wastewater-based surveillance in a region was assessed with regard to its reliability as a follow-up method and its possible use as a predictive tool to anticipate epidemiological dynamics. Although the virologic determinations were robust, the main limitation was the difficulty of a daily analysis that could be used for more precise estimations and predictions. In the present work, the measurements of 32 WWTPs, covering a population of 4,171,668 inhabitants, were analysed. The sampling periodicity was weekly at all the studied stations. From a practical point of view, the main limitations for more frequent sampling were related to personnel availability, especially in the pandemic context. In situations of epidemiological crises, once the stations that show this correlation between concentration and incidence have been selected, it should be possible to perform measurements with a higher frequency, probably a daily measurement. This would increase the prediction potential based on WWTP sampling.

Nevertheless, working with weekly measurements will be of particular interest in situations where the incidence is low and in the initial and final stages when epidemiological data are limited. Moreover, WWTP-based surveillance with a weekly sample can be of interest in certain countries or regions where epidemiological surveillance presents strong limitations. In these situations, wastewater can be a good alternative to build a picture of the actual epidemiological situation. Moreover, the switch of COVID-19 surveillance from a global testing strategy (i.e., testing both symptomatic and asymptomatic) to a sentinel-based system (testing of serious cases together with a sentinel system to monitor global transmission) can benefit from the reinforcement of WWTP-based surveillance combined with a prediction system.

When attempting to relate WWTP-based surveillance with epidemiological curves, two types of behaviours were found. In 68.8% of the studied contagion waves (2 contagion waves in 32 WWTPs), there was a good correlation between RNA concentrations in water and the incidence within the area of water collection, while the correlation was poor at other stations. This observation suggests that, at some wastewater stations, there is no clear relationship between the real source of such wastewater and the population that is assigned to the corresponding health area that will confer the health surveillance database. Several factors may explain such behaviours, mostly citizens' mobility due to the socioeconomic characteristics of the municipalities corresponding to each WWTP and its associated BHAs. Note that BHAs where disease cases are reported are assigned according to citizens’ home address, while contribution to sewage water by these same citizens may occur at different locations (mostly at workplace or at holiday destination), possibly associated with a WWTP different than that corresponding to the BHA.

This could explain the observed mismatch between WWTP-based and BHA-based surveillance. For instance, in mainly residential municipalities, the relationship between WWTP virus concentration and reported cases may be lost if the workplace of the residents is mostly located in municipalities associated with other WWTPs. Similarly, in municipalities with important industrial or service activities, infected workers and customers from other municipalities will contribute to the corresponding WWTP virus concentration and later report their cases to unrelated BHAs, again breaking the relationship between reported cases and sewage water concentration. These behaviours may also have a strong seasonal component (due to tourism, holidays, second homes, etc.) and are also affected by the nonpharmacological measures imposed by the competent authorities (forced teleworking, closing or restriction of catering schedules, etc.).

It was beyond the scope of this study to analyse the particular behaviours of each WWTP and corresponding BHAs, and the results focused only on identifying and quantifying those sites where the case/concentration relationship held. In fact, it has been shown that, using the set of stations and the corresponding population as a whole, the correlation between concentration and incidence is satisfactory, which is independent of the movements inside the global region. If effective WWTP-based monitoring is to be carried out at a local scale, it is necessary to select the stations that show a good relationship between the population that dumps biological waste and the population that remains under the control of the health system.

An important result was the definition and validation of the mathematical function that relates RNA concentration in water and COVID-19 incidence. This feature was selected after evaluating multiple alternatives. Although the function developed requires calibration for each of the stations, the calibration process is reasonable, and a range of results was clearly established. Furthermore, the empirical nature of the approach does not rely on ad-hoc hypotheses that may be particular of a certain spatio-temporal context, for instance, given a particular variant or setting. This makes it possible to assess incidence intervals at stations where there is no good epidemiological follow-up. This is an especially important alternative for cities in countries with low or medium HDIs that have wastewater collection but do not have a good epidemiological monitoring system. In these cases, and with the results shown, it is feasible to use only the monitoring of wastewater to know the evolution of the virus among the population. For this, it is recommended to analyse wastewater at least once a week, and to have the accumulated incidence during a wave for the calibration of the model.

## Supplementary Information

Supplementary Information.

## Data Availability

All the data used in the present work can be freely accessed. Cumulative incidence data is provided in https://analisi.transparenciacatalunya.cat. SARS-CoV-2 concentration in wastewater samples is provided in https://sarsaigua.icra.cat.
