# Supplementary material for: Assessing wastewater-based epidemiology for the prediction of SARS-CoV-2 incidence in Catalonia
Source: Sci Rep. 2022 Sep 5;12:15073. doi: 10.1038/s41598-022-18518-9 (PMC9443647; doi:10.1038/s41598-022-18518-9)
Supplement: Supplementary file 1 — Supplementary Information. [file 41598_2022_18518_MOESM1_ESM.docx]

## Assessing Wastewater-based Epidemiology for the prediction of SARS-CoV-2 incidence in Catalonia

Bernat Joseph-Duran^1, *^, Albert Serra-Compte^1^, Miquel Sàrrias^1^, Susana Gonzalez^1^,

Daniel López^2^, Clara Prats^2^, Martí Català^2^, Enric Álvarez^2^, Sergio Alonso^2^, Marina Arnaldos^1^

^1^ CETAQUA Water Technology Center, Cornellà de Llobregat, Catalonia, Spain.

^2^ Department of Physics, Universitat Politècnica de Catalunya (UPC-BarcelonaTech), Barcelona, Catalonia, Spain.

* [bjoseph@cetaqua.com](mailto:bjoseph@cetaqua.com)

## Supplementary information

Table 1. Characteristics of the targeted WWTPs.

| WWTP Name | WWTP ID | Population (of corresponding BHAs) [Inhab.] | WWTP Design Capacity [Inhab. Eq.] | Integrated Sampling Time [hours] | Wave Model Autumn Mean R2 | Wave Model Winter Mean R2 | Linear Regression Forecast Mean MAPE [%] | Wave Model Forecast Mean MAPE [%] |
| --- | --- | --- | --- | --- | --- | --- | --- | --- |
| Abrera | WWTP1 | 63913 | 115000 | 24h | 0.93 | 0.8 | 11.83 | 29.08 |
| Amposta | WWTP2 | 51519 | 27500 | 24h | 0.85 | 0.91 | 48.29 | 32.72 |
| Balaguer | WWTP3 | 28076 | 18750 | 24h | 0.99 | 0.82 | 16.7 | 26.41 |
| Banyoles | WWTP4 | 47823 | 186560 | 24h | 0.91 | 0.75 | 12.88 | 22.18 |
| Berga | WWTP5 | 21536 | 41500 | 24h | 0.97 | 0.74 | 25.73 | 31.97 |
| Besòs | WWTP6 | 1605816 | 3000000 | 24h | 0.04 | 0.85 | 10.41 | 42.31 |
| Figueres | WWTP7 | 63886 | 110640 | 24h | 0.91 | 0.78 | 14.21 | 38.87 |
| Girona | WWTP8 | 148911 | 206250 | 24h | 0.92 | 0.53 | 7.82 | 17.84 |
| Gavà - Viladedcans | WWTP9 | 24164 | 300000 | 24h | 0.67 | 0.97 | 19.42 | 36.1 |
| Igualada | WWTP10 | 74945 | 285666 | 24h | 0 | 0.89 | 15.73 | 30.79 |
| Lleida | WWTP11 | 174733 | 186000 | 24h | 0.86 | 0 | 13.13 | 25.9 |
| Manlleu | WWTP12 | 37730 | 44153 | 24/16h | 0.01 | 0.23 | 12.47 | 28.66 |
| Manresa | WWTP13 | 104755 | 196167 | 24h | 0.07 | 0.59 | 8.3 | 29.02 |
| Mataró | WWTP14 | 188317 | 451250 | 24h | 0.86 | 0.73 | 14.09 | 50.85 |
| Montcada | WWTP15 | 143432 | 423500 | 24h | 0.92 | 0.55 | 7.93 | 26.28 |
| Montferrer | WWTP16 | 16222 | 25000 | 8h | 0.63 | 0.96 | 18.09 | 25.44 |
| Martorell | WWTP17 | 29586 | 61250 | 24h | 0.92 | 0.89 | 23.33 | 28.26 |
| Olot | WWTP18 | 44782 | 99166 | 24h | 0.29 | 0.94 | 14.4 | 47.45 |
| Palamós | WWTP19 | 28960 | 165450 | 24h | 0.62 | 0.74 | 21.72 | 28.2 |
| El Prat de Llobregat | WWTP20 | 30140 | 2000000 | 24h | 0.97 | 0.86 | 15.89 | 26.28 |
| Ripoll | WWTP21 | 20214 | 45000 | 24h | 0.62 | 0.42 | 16.63 | 83.76 |
| Rubí | WWTP22 | 88765 | 135000 | 24h | 0.85 | 0.88 | 15.85 | 14.98 |
| Reus | WWTP23 | 119664 | 195833 | 24h | 0.77 | 0.94 | 35.51 | 44.52 |
| Sant Feliu de Llobregat | WWTP24 | 216253 | 320000 | 24h | 0.6 | 0.7 | 8.34 | 29.94 |
| Sabadell Riu Sec | WWTP25 | 30823 | 200000 | 24h | 0.82 | 0.71 | 12.38 | 28.4 |
| Tarragona | WWTP26 | 161291 | 175000 | 12h | 0.25 | 0.76 | 22.34 | 67.04 |
| Tortosa - Roquetes | WWTP27 | 46618 | 46847 | 24h | 0.91 | 0.96 | 32.32 | 31.21 |
| Terrassa | WWTP28 | 244984 | 400000 | 24h | 0.44 | 0.71 | 11.61 | 36.21 |
| Vilafranca del Penedès | WWTP29 | 75558 | 192000 | 24h | 0.91 | 0.9 | 14.26 | 24.4 |
| Riera de la Bisbal | WWTP30 | 62354 | 100000 | 24h | 0.78 | 0.58 | 23.07 | 40.03 |
| Vic | WWTP31 | 71091 | 204166 | 24h | 0.23 | 0.76 | 15.19 | 35.16 |
| Vilanova i la Geltrú | WWTP32 | 104807 | 153000 | 24h | 0.96 | 0.86 | 17.86 | 37.29 |
